# Brain tissue oxygen monitoring in traumatic brain injury—part II: isolated and combined insults in relation to outcome

**DOI:** 10.1186/s13054-023-04659-4

**Published:** 2023-09-26

**Authors:** Teodor Svedung Wettervik, Erta Beqiri, Anders Hånell, Stefan Yu Bögli, Michal Placek, Mathew R. Guilfoyle, Adel Helmy, Andrea Lavinio, Ronan O’Leary, Peter J. Hutchinson, Peter Smielewski

**Affiliations:** 1 Department of Medical Sciences, Section of Neurosurgery, Uppsala University, 751 85 Uppsala, Sweden; 2 Brain Physics Laboratory, Department of Clinical Neurosciences, Division of Neurosurgery, University of Cambridge, Cambridge, UK; 3 Division of Neurosurgery, Department of Clinical Neurosciences, Addenbrooke’s Hospital, University of Cambridge, Cambridge, UK; 4 Neurosciences and Trauma Critical Care Unit, Addenbrooke’s Hospital, Cambridge University Hospitals, Cambridge, UK

**Keywords:** Brain tissue oxygenation, Neurocritical care, Outcome, Thresholds, Traumatic brain injury

## Abstract

**Background:**

The primary aim was to explore the concept of isolated and combined threshold-insults for brain tissue oxygenation (pbtO_2_) in relation to outcome in traumatic brain injury (TBI).

**Methods:**

A total of 239 TBI patients with data on clinical outcome (GOS) and intracranial pressure (ICP) and pbtO_2_ monitoring for at least 12 h, who had been treated at the neurocritical care unit, Addenbrooke’s Hospital, Cambridge, UK, between 2002 and 2022 were included. Outcome was dichotomised into favourable/unfavourable (GOS 4–5/1–3) and survival/mortality (GOS 2–5/1). PbtO_2_ was studied over the entire monitoring period. Thresholds were analysed in relation to outcome based on median and mean values, percentage of time and dose per hour below critical values and visualised as the combined insult intensity and duration.

**Results:**

Median pbtO_2_ was slightly, but not significantly, associated with outcome. A pbtO_2_ threshold at 25 and 20 mmHg, respectively, yielded the highest *x*^2^ when dichotomised for favourable/unfavourable outcome and mortality/survival in chi-square analyses. A higher dose and higher percentage of time spent with pbtO_2_ below 25 mmHg as well as lower thresholds were associated with unfavourable outcome, but not mortality. In a combined insult intensity and duration analysis, there was a transition from favourable towards unfavourable outcome when pbtO_2_ went below 25–30 mmHg for 30 min and similar transitions occurred for shorter durations when the intensity was higher. Although these insults were rare, pbtO_2_ under 15 mmHg was more strongly associated with unfavourable outcome if, concurrently, ICP was above 20 mmHg, cerebral perfusion pressure below 60 mmHg, or pressure reactivity index above 0.30 than if these variables were not deranged. In a multiple logistic regression, a higher percentage of monitoring time with pbtO_2_ < 15 mmHg was associated with a higher rate of unfavourable outcome.

**Conclusions:**

Low pbtO_2_, under 25 mmHg and particularly below 15 mmHg, for longer durations and in combination with disturbances in global cerebral physiological variables were associated with poor outcome and may indicate detrimental ischaemic hypoxia. Prospective trials are needed to determine if pbtO_2_-directed therapy is beneficial, at what individualised pbtO_2_ threshold therapies are warranted, and how this may depend on the presence/absence of concurrent cerebral physiological disturbances.

## Introduction

Neurocritical care (NCC) aims to reduce secondary insults and thereby avoid development of secondary brain injury in traumatic brain injury (TBI) [1]. Traditional NCC has focused on treating elevated intracranial pressure (ICP) and keeping cerebral perfusion pressure (CPP) sufficiently high to avoid secondary brain injury [2]. However, in addition to ICP and CPP, there has been an increased understanding of the complex interplay of other pathophysiological mechanisms in TBI such as disrupted cerebral pressure autoregulation, microvascular thrombosis, and oxygen diffusion limitations [1, 3–5]. Brain tissue oxygenation (pbtO_2_) is a downstream variable to CBF and focal pbtO_2_ monitoring has gained increased interest in NCC of TBI patients [1]. Several smaller studies indicate that low pbtO_2_ correlates with worse clinical outcome, although the “optimal” pbtO_2_ threshold for outcome prognosis has varied between approximately 10 to 30 mmHg in these studies [6–10]. Low pbtO_2_ was initially thought to primarily reflect ischaemic hypoxia. However, it seems that low pbtO_2_ often occurs despite normal perfusion-related variables such as ICP and CPP [6, 9] and it has then been suggested to rather indicate other pathophysiological mechanisms such as oxygen diffusion limitations [11, 12].

More recently, integrated management protocols with pbtO_2_ monitoring have been explored to better determine when increased cerebral oxygen delivery is needed [13, 14]. A recent phase II trial showed a trend towards lower mortality for patients treated with a combined protocol of ICP and pbtO_2_-therapy as compared to ICP therapy alone [14]. There are now three ongoing phase III trials (BOOST-3, OXY-TC, and BONANZA) on pbtO_2_-directed therapies in TBI, which will reveal if such therapy benefits patient recovery [13]. However, to better understand the prognostic implications of pbtO_2_ and how to use this tool for clinical decision-making, it is of value to further explore the critical pbtO_2_ threshold values in relation to outcome.

In part I of this study, we explored the associations between global cerebral physiological variables (e.g. ICP and CPP) in relation to pbtO_2_. We found that pbtO_2_ below 20 mmHg was relatively frequent, but this was often an independent event and only weakly associated with these global physiological variables. Considering this weak association, the prognostic value of pbtO_2_ remains to be determined and how this depends on concurrent disturbances in the global cerebral physiological variables. In part II of this study (current article), we aimed to investigate different definitions of pbtO_2_ threshold-insults in relation to clinical outcome in TBI. We also aimed to determine whether concurrent disturbances in global cerebral physiological variables influenced the relation between pbtO_2_ and outcome. We hypothesised that pbtO_2_ at 25 mmHg and lower for longer episodes of time would be associated with worse outcome and that the association between low pbtO_2_ and outcome would be more pronounced when global cerebral physiological variables such as ICP and CPP were also deranged.

## Materials and methods

### Patients, study design, and management protocol

In this observational, single-centre study, 781 de-identified records of high-resolution neuromonitoring data of TBI patients admitted in the NCC unit of the Addenbrooke’s Hospital (Cambridge, UK) between 2002 and 2022, were screened from the Brain Physics Lab research database. All patients had ABP and ICP data, 461 of these patients were also monitored with pbtO_2_, but those 36 who had less than 12 h of available ICP and pbtO_2_ data and those 186 without available outcome data were excluded. Thus, 239 patients were included in the final patient population, and they were admitted between 2006 and 2022. Ten-second averages of pre-processed cleaned ABP, ICP and pbtO_2_ data were accessed. The following clinical descriptors were also retrieved: age, sex, Glasgow Coma Scale (GCS), pupillary reactivity, decompressive craniectomy, and Glasgow Outcome Scale (GOS).

The management protocol has been described in detail in part I as well as in previous studies [15, 16]. In brief, CPP was targeted above 60 mmHg, ICP below 20 to 22 mmHg, pbtO_2_ above 20 mmHg, partial pressure of carbon dioxide within 4.5–5 kPa, and arterial glucose within 6 to 8 mmol/L. The pressure reactivity index (PRx) and optimal CPP (CPPopt) were introduced at the bedside in 1999 and 2012, respectively, and were primarily used as monitoring variables. PbtO_2_ was mainly maintained above 20 mmHg by reducing ICP and increasing CPP. Additional efforts to keep pbtO_2_ above the designated target was allowed based on the treating physician's discretion. This could involve raising the fraction of inspired oxygen (FiO_2_) levels or implementing a moderate upward adjustment of CPP.

### Clinical outcome and physiological measurements

During the study period of 20 years, GOS was used for outcome evaluation in the early years and GOS-E in the later years. To make the analyses consistent for all patients with outcome data, we converted GOS-E to GOS scores. GOS/GOS-E was evaluated at 6 months post-injury, either as a clinical assessment or via telephone interviews by trained staff. GOS ranges from 1 (death) and 5 (good recovery) [17, 18]. Outcome was dichotomised as favourable/unfavourable (GOS 4–5/1–3) and survival/mortality (GOS 2–5/1).

Collection of physiological data including ICP, ABP, and pbtO_2_ and the data processing of these variables were described in detail in part I. ICM + (ICM + software, Cambridge Enterprises, University of Cambridge, UK) [https://icmplus.neurosurg.cam.ac.uk] was used for physiological measurements data processing. PRx was calculated as the moving Pearson correlation coefficient of 30 consecutive 10-s average values of ABP and ICP and updated every minute [19, 20]. Minute-by-minute values of CPPopt were calculated as the CPP with the concurrently lowest PRx, based on the multi-window weighted algorithm based on a data buffer of 2 to 8 h [21].

### Isolated pbtO_2_ insults definitions

The insults of pbtO_2_ were defined based on five different methods and analysed in relation to favourable/unfavourable outcome and survival/mortality. The first definitions were cruder, whereas the latter allowed for more granular ways to study pbtO_2_ insults. In the first insult definition, differences in median and mean values over the entire monitoring time were tested in relation to the outcome groups. In the second definition, we used a similar approach as Sorrentino et al. [22] and explored a range of thresholds (below 10, 15, 20, 25, 30, 35, and 40 mmHg) and then dichotomised the patients based on if their median value for that variable was above or below the specific threshold. Thus, we chose pbtO_2_ values from the normal to lower range and divided it into 5 mmHg-intervals. These dichotomisations were compared to the patients’ outcome (favourable/unfavourable and survival/mortality) using a chi-square test. The threshold with the highest x^2^ was considered as optimal for outcome dichotomisation. In the third insult definition, the percentage of good monitoring time (%GMT, i.e. the remaining data after cleaning) below the same critical values as mentioned above was calculated for pbtO_2_ (below 10, 15, 20, 25, 30, 35, and 40 mmHg) [23]. In the fourth insult definition, the dose (area under the curve based on hours below the threshold combined with the absolute values below that value) per hour was calculated for the same thresholds that were explored above [24]. In the fifth insult definition, insult intensity (mmHg) and duration (minutes) were assessed and visualised using the method originally described by Guïza and colleagues [25, 26], which was implemented and adapted by the Uppsala group in a custom-written R-script [27–29]. For each combination of intensity and duration, the correlation with outcome was determined as the average number of insults for every GOS category (1 to 5) [25, 26]. Positive correlation coefficients indicated an association with favourable outcome for that pixel and negative correlation coefficients indicated an association with unfavourable outcome. Gaussian smoothing with a standard deviation of 1 was applied to the matrix of correlation values to reduce high-frequency noise. The final correlation values were visualised using the jet colour scale. Pixels with correlation coefficients at + 1 were coloured blue and -1 were coloured red, where red indicates stronger correlation with mortality. Two important modifications were added to the original algorithm proposed by Guïza et al.:

1. Regions with insufficient data (less than 20 patients per pixel/correlation) were not included in the maps, i.e. they were coloured white.
2. For each pixel of the insults image, the duration was not cumulative, i.e. an insult with e.g. pbtO_2_ below 15 mmHg for 20 min was specifically counted as one insult only for the pixel representing 20 min, but was not counted as an accumulated insult in pixels with lower durations that had already been exceeded (e.g. 19 min). The intensity was still cumulative in this study.

In addition, prior to analysis, shorter gaps in the data (10 min or less) were closed via a linear approximation between the value just prior to and after the gap. The gaps were closed to avoid breaking up longer insults because of a small number of missing values. Insults where the duration was determined by a gap in the data, or the start or end of the recording were excluded from the analysis since their true duration could not be determined.

### Combined pbtO_2_ insults definitions

The %GMT was calculated for pbtO_2_ below 15 mmHg (the %GMT threshold most strongly associated with unfavourable outcome as an isolated insult) in combination with other perfusion-related variables: ICP > 20 mmHg, PRx > 0.30, CPP < 60 mmHg, or ∆CPPopt < −5 mmHg. The ICP and CPP thresholds were chosen in accordance with the management protocol. Although PRx is not a direct measure of brain perfusion, we chose to include this variable since positive PRx indicates that the vascular reactivity is impaired and the brain is deprived of the protective mechanism that could stabilise CBF over a certain range of CPP and thus prevent ischaemia or hyperaemia. We chose PRx above 0.30, which has previously been strongly associated with poor outcome and may reflect approximately when the limit of autoregulation is exceeded [22, 30]. ∆CPPopt < −5 mmHg was previously suggested as the autoregulatory threshold for hypoperfusion [31].

In addition, the interactions of pbtO_2_ with ICP, PRx, CPP, and ∆CPPopt, respectively, in relation to outcome were visualised using the metric of %GMT fulfilling both thresholds criteria (for pbtO_2_ and for the other variable) in relation to GOS in heatmaps [32]. The heatmaps were created using a custom-written R-script. PbtO_2_ (range 5 to 40, 1 mmHg resolution) together with ICP (range 0–40 mmHg, 1 mmHg resolution), PRx (range -1–1, 0.05 resolution), CPP (range 40 to 100 mmHg, 2 mmHg resolution), and ∆CPPopt (range -30 to + 30 mmHg, 2 mmHg resolution) yielded a grid of 1 400 cells (35 * 40) for the plots with pbtO_2_ vs ICP- and PRx and 1 050 cells (35 * 30) for the two other plots. For each coordinate/pixel (combination of two thresholds) the %GMT was calculated for all patients and correlated with GOS using the Spearman test. To produce smoother images, each pixel was divided into 3 by 3 subpixels followed by application of a Gaussian kernel filter (standard deviation of 2 pixels). The final values for each pixel were then translated into the jet colour scale (red to blue) with red/blue colour indicating an association with unfavourable/favourable outcome (GOS). Due to the moderate correlation strength (r within ± 0.3), the jet colour scale was limited to this correlation coefficient range. Pixels with less than 5 patients with at least 5 min of monitoring time for the corresponding combination of pbtO_2_ with ICP, PRx, CPP, or ∆CPPopt were excluded, i.e. coloured as white. In addition, a density plot was generated to visualise the frequency of the %GMT for certain combinations of pbtO_2_ with ICP, PRx, CPP, or ∆CPPopt. The resulting numbers were normalised (divided) by the highest count within the grid to yield density values ranging from 0 to 1 for each cell in the grid. A Gaussian smoothing, similar to the above procedure, was applied here as well and the final values were then transformed to colours using the jet colour scale.

### Statistical analysis

The statistical analyses were conducted in RStudio software (version 2022.12.0) [33]. Continuous/ordinal variables were described as medians (interquartile range (IQR)) and categorical variables as numbers (proportions). The association of pbtO_2_ insults (median and mean values, %GMT and dose per hour below threshold) with favourable/unfavourable outcome and survival/mortality was analysed with the Mann—Whitney U test and/or the chi-square test. Similar outcome analyses were done with the pbtO_2_-%GMT threshold most strongly associated with outcome in combination with ICP > 20 mmHg, PRx > 0.30, CPP < 60 mmHg, and ∆CPPopt < −5 mmHg. Multiple logistic outcome regressions were conducted with unfavourable outcome and mortality, respectively, as the dependent variable. Age and GCS together with the %GMT of ICP > 20 mmHg, PRx > 0.30, CPP < 60 mmHg, and the most significant pbtO_2_ threshold from the univariate insult-analyses were used as independent variables in these regressions. In similar, but separate regressions of unfavourable outcome and mortality, the %GMT of ∆CPPopt < −5 mmHg replaced CPP < 60 mmHg as an independent variable, to determine the role of the autoregulatory rather than the absolute, fixed CPP targets. A p-value below 0.05 was considered statistically significant. We abstained from adjustment for multiple testing since this was an exploratory study and many of the significance tests overlapped to a high degree.

## Results

### Demographics, clinical variables, treatments, and outcome

In this TBI cohort of 239 patients (Table 1), the median age was 35 (IQR 25–55) years and the male/female ratio was 176/45 (80/20%). The median GCS immediately after the trauma was 7 (IQR 4–9) and pupillary reactivity was preserved in 135 (85%), unilaterally unreactive in 18 (11%), and bilaterally unreactive in 5 (3%) patients. Fifty-seven (27%) patients underwent decompressive craniectomy during NCC. At 6 months of follow-up, the median GOS was 4 (IQR 2–4), 124 (52%) had recovered favourably, and 58 (24%) were deceased. The clinical data were missing in some patients (Table 1).

**Table 1:** Demographics, admission variables, treatments, and clinical outcome

Patients, *n* (%)	239 (100%)
Age (years), median (IQR)	35 (25–55)
Sex (male/female), *n* (%)	176/45 (80/20%)
GCS (scale), median (IQR)	7 (4–9)
Pupillary reactivity (normal/1 unreactive/2 unreactive), *n* (%)	135/18/5 (85/11/3%)
Decompressive craniectomy, *n* (%)	57 (27%)
GOS, median (IQR)	4 (2–4)
Favourable/unfavourable outcome, *n* (%)	124/115 (52/48%)
Mortality, *n* (%)	58 (24%)

Number of patients with missing data: age (*n* = 3), sex (*n* = 18), GCS (*n* = 18), pupillary reactivity (*n* = 81), decompressive craniectomy (*n* = 30)

*GCS* Glasgow Coma Scale, *GOS* Glasgow Outcome Scale, *IQR* interquartile range

### Cerebral physiological variables during neurocritical care

During NCC, the median ICP was 13 (IQR 10–17) mmHg, the median PRx was 0.01 (IQR −0.08–0.15), the median CPP was 75 (IQR 72–80) mmHg, the median ∆CPPopt was 0 (IQR -1–2) mmHg, and the median pbtO_2_ was 27 (IQR 21–33) mmHg. The median number of days with available data of ICP and pbtO_2_ from the NCC was 5 (IQR 3–8) and 5 (2–7) days, respectively. The median percentage of time with available pbtO_2_ and ICP data during the NCC was 69% (IQR 45–85) and 77% (IQR 60–89), respectively.

### PbtO_2_ insults in relation to favourable outcome and mortality

**Fig. 1:**
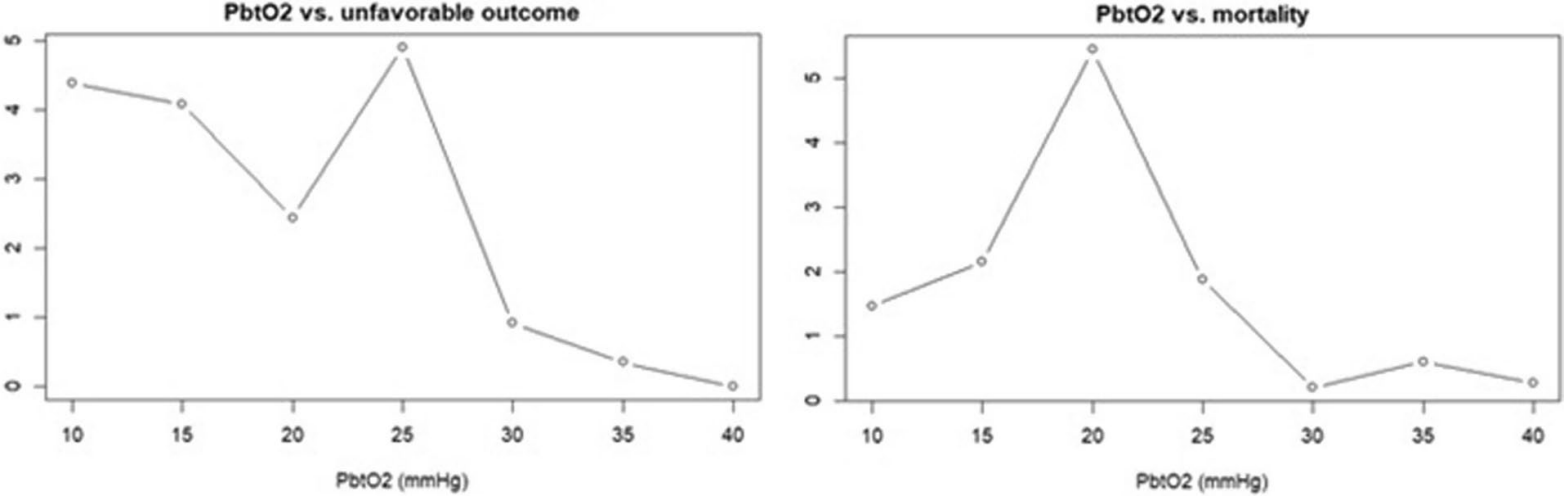
PbtO_2_ in relation to unfavourable outcome and mortality—a chi-square analysis. The figure illustrates that the pbtO_2_ threshold at 25 and 20 mmHg, respectively, exhibited the highest/best *x*^2^ for favourable/unfavourable outcome and mortality/survival. PbtO_2_ = Partial brain tissue oxygenation

**Fig. 2:**
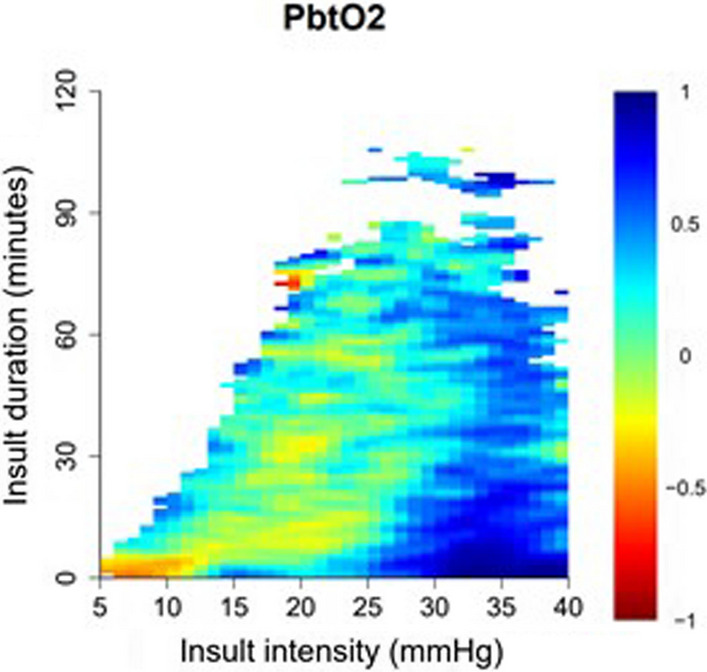
PbtO_2_-insult intensity and duration in relation to clinical outcome. The plot was more frequently white in the left corner due to the limited number of patients with such long and severe insults. The plot was also white in the right corner, since these low intensity insults were typically either very brief or long. As illustrated, there was a transition from better to worse outcome when pbtO_2_ went below 20 to 30 mmHg. PbtO_2_ = Partial brain tissue oxygenation

Mean (but not median) pbtO_2_ was significantly higher in patients with favourable as compared to unfavourable outcome, butthere was no difference related to mortality (Table 2). Using the chi-square approach, a pbtO_2_ threshold at 25 and 20 mmHg yielded the highest *x*^2^ (Fig. 1), when dichotomised for favourable/unfavourable outcome and mortality/survival, respectively. A higher percentage of monitoring time with pbtO_2_ below 25 mmHg and lower thresholds were associated with unfavourable outcome. A higher dose of pbtO_2_ below 40 mmHg and lower thresholds were also associated with unfavourable outcome (Table 2). A pbtO_2_ threshold at 15 mmHg had the strongest association with unfavourable outcome using both the %GMT and the dose concept. Neither the %GMT nor the dose of pbtO_2_ below threshold were associated with mortality. In a combined insult intensity and duration analysis (Fig. 2), there was a transition from favourable towards unfavourable outcome when pbtO_2_ went below 25–30 mmHg for 30 min and similar was true for shorter durations when the intensity was higher.

**Table 2:** PbtO_2_-insult definitions in relation to favourable outcome and mortality

	All	Favourable	Unfavourable	*p*	Survivor	Deceased	*p*
Median pbtO_2_	27 (21–33)	28 (21–30)	25 (20–31)	0.06	27 (22–33)	25 (19–31)	0.09
Mean pbtO_2_	27 (22–33)	28 (23–34)	25 (21–32)	***0.045***	28 (22–34)	25 (19–31)	0.07
*%GMT below threshold*							
pbtO_2_ < 10 mmHg (%)	0 (0–2)	0 (0–1)	0 (0–4)	***0.008***	0 (0–1)	0 (0–4)	0.17
pbtO_2_ < 15 mmHg (%)	3 (0–15)	2 (0–9)	6 (0–19)	***0.008***	3 (0–13)	5 (0–22)	0.11
pbtO_2_ < 20 mmHg (%)	17 (4–43)	12 (2–40)	22 (4–53)	***0.02***	15 (3–41)	21 (4–56)	0.13
pbtO_2_ < 25 mmHg (%)	43 (14–74)	35 (10–66)	51 (19–76)	***0.046***	40 (14–69)	52 (14–85)	0.12
pbtO_2_ < 30 mmHg (%)	69 (39–93)	64 (31–89)	79 (46–96)	0.051	67 (35–91)	80 (45–97)	0.08
pbtO_2_ < 35 mmHg (%)	89 (63–99)	84 (58–98)	92 (65–99)	0.06	86 (62–98)	92 (71–100)	0.09
pbtO_2_ < 40 mmHg (%)	97 (79–100)	96 (79–100)	98 (81–100)	0.12	97 (79–100)	98 (87–100)	0.14
*Dose below threshold per hour*							
pbtO_2_ < 10 mmHg (%)	0.0 (0.0–0.0)	0.0 (0.0–0.0)	0.0 (0.0–0.1)	***0.01***	0.0 (0.0–0.0)	0.0 (0.0–0.1)	0.15
pbtO_2_ < 15 mmHg (%)	0.1 (0.0–0.4)	0.0 (0.0–0.2)	0.1 (0.0–0.5)	***0.006***	0.0 (0.0–0.3)	0.1 (0.0–0.5)	0.13
pbtO_2_ < 20 mmHg (%)	0.5 (0.1–1.8)	0.4 (0.1–1.4)	0.9 (0.1–2.4)	***0.008***	0.5 (0.1–1.5)	0.6 (0.1–2.6)	0.11
pbtO_2_ < 25 mmHg (%)	2.0 (0.6–4.9)	1.6 (0.4–4.1)	2.4 (0.8–5.6)	***0.01***	1.8 (0.6–4.3)	2.4 (0.6–6.4)	0.13
pbtO_2_ < 30 mmHg (%)	4.8 (2.0–8.8)	3.9 (1.4–7.9)	5.7 (2.5–10.0)	***0.01***	4.5 (2.0–8.4)	5.9 (2.0–11.1)	0.11
pbtO_2_ < 35 mmHg (%)	8.6 (4.4–13.5)	7.6 (3.7–12.7)	10.2 (5.3–14.8)	***0.02***	10.1 (5.3–14.8)	10.0 (4.9–15.9)	0.10
pbtO_2_ < 40 mmHg (%)	13.1 (8.5–18.5)	12.2 (7.5–17.6)	14.8 (9.4–19.6)	***0.03***	14.8 (9.5–19.6)	14.6 (9.2–20.9)	0.09

Bold and italics indicate statistical significance

The variables in the table were described as median values (IQR). The Mann–Whitney *U* test was used to compare pbtO_2_ in the different outcome groups

*GMT* good monitoring time, *IQR* interquartile range, *PbtO*_*2*_ partial brain tissue oxygenation

### Combined insults of pbtO_2_ together with disturbances in ICP, PRx, CPP, or ∆CPPopt

**Fig. 3:**
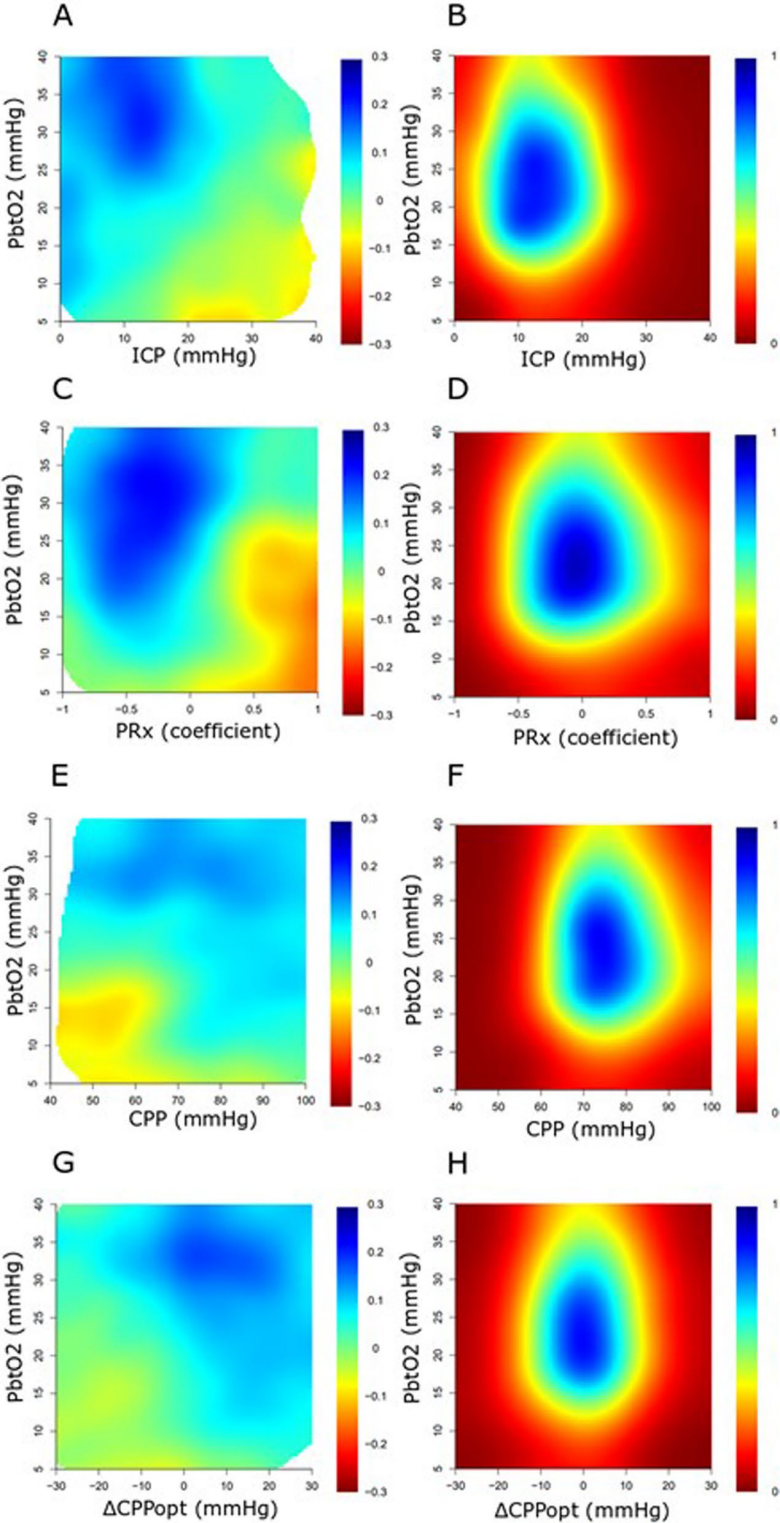
A-H. PbtO_2_ in combination with ICP, PRx, CPP, and ∆CPPopt—relation to clinical outcome. In (**A**), the percentage of monitoring time for the concurrent combination of pbtO_2_ and ICP was calculated and correlated with GOS. The jet colour range denotes the value of the correlation coefficients, where blue colour indicates association with favourable and red colour indicates association with unfavourable outcome. Areas with limited data were given white colour. In (**B**), the density plot illustrates the data frequency of certain pbtO_2_ and ICP combinations. The jet colour scale denotes the frequency, where blue colour indicates that a certain pbtO_2_/ICP combination was common, whereas red colour indicates that a certain pbtO_2_/ICP combination was rare. Similar analyses were done for pbtO_2_ and PRx in relation to GOS (**C**, **D**), pbtO_2_ and CPP in relation to GOS (**E**, **F**), and pbtO_2_ and ∆CPPopt in relation to GOS (**G**, **H**). CPP = Cerebral perfusion pressure. CPPopt = Optimal CPP. GOS = Glasgow Outcome Scale. ICP = Intracranial pressure. PbtO_2_ = Partial brain tissue oxygenation. PRx = Pressure reactivity index. ∆CPPopt = Actual CPP-CPPopt

A higher %GMT of ICP above 20 mmHg was associated with mortality, and this was more pronounced when pbtO_2_ was below rather than above 15 mmHg (Table 3). Furthermore, a higher %GMT of PRx above 0.30 was associated with unfavourable outcome and mortality, which was more pronounced for unfavourable outcome when pbtO_2_ was below rather than above 15 mmHg. In addition, a higher %GMT of CPP below 60 mmHg was associated with unfavourable outcome and mortality and this was more pronounced when pbtO_2_ was below than above 15 mmHg. Lastly, a higher %GMT with ∆CPPopt below -5 mmHg was associated with both unfavourable outcome and mortality, which was more pronounced, but still only marginally significant when pbtO_2_ was below rather than above 15 mmHg. Overall, pbtO_2_ below 15 mmHg together with disturbances in any of the global physiological variables, i.e. ICP > 20 mmHg, PRx > 0.30, CPP < 60 mmHg, or ∆CPPopt < −5 mmHg, occurred in median during slightly below 1% of GMT. Furthermore, a higher %GMT of pbtO_2_ below 15 mmHg without any concurrent disturbance in any of the global cerebral physiological variables was not associated with unfavourable outcome or mortality. Figure 3 also illustrates that the combination of lower pbtO_2_ with higher ICP, higher PRx, lower CPP, and more negative ∆CPPopt was associated with worse clinical outcome than isolated insults in any of these variables.

**Table 3:** Insult-combinations of pbtO_2_ and ICP, PRx, CPP, and ∆CPPopt in relation to favourable outcome and mortality

	All	Favourable	Unfavourable	*p*	Survivor	Deceased	*p*
*ICP and pbtO*_*2*_*, %GMT above/below threshold*							
ICP > 20 mmHg (%)	9 (2–32)	7 (2–28)	14 (2–36)	0.15	7 (2–28)	28 (5–58)	** < *0.001***
ICP > 20 mmHg and pbtO_2_ < 15 mmHg (%)	0.2 (0.0–1.4)	0.2 (0.0–0.7)	0.2 (0.0–2.1)	0.11	0.1 (0.0–0.8)	0.8 (0.0–5.4)	***0.007***
ICP > 20 mmHg and pbtO_2_ > 15 mmHg (%)	7 (2–22)	7 (2–20)	9 (1–23)	0.64	6 (2–18)	14 (3–34)	***0.01***
ICP < 20 mmHg and pbtO_2_ < 15 mmHg (%)	1 (0–8)	1 (0–7)	2 (0–10)	0.55	1 (0–10)	1 (0–8)	0.39
*PRx and pbtO*_*2*_*, %GMT above/below threshold*							
PRx > 0.30 (%)	25 (17–36)	22 (16–32)	29 (20–48)	** < *0.001***	22 (16–32)	39 (26–64)	** < *0.001***
PRx > 0.30 and pbtO_2_ < 15 mmHg (%)	0.5 (0.0–2.5)	0.4 (0.0–1.6)	0.9 (0.1–4.1)	***0.02***	0.4 (0.0–1.9)	1.6 (0.1–6.1)	***0.02***
PRx > 0.30 and pbtO_2_ > 15 mmHg (%)	18 (11–27)	18 (12–23)	19 (11–30)	0.26	17 (11–23)	24 (15–44)	** < *0.001***
PRx < 0.30 and pbtO_2_ < 15 mmHg (%)	0.9 (0.0–5.7)	1.0 (0.0–5.2)	0.8 (0.0–6.3)	0.68	1.0 (0.0–6.2)	0.8 (0.1–5.5)	0.92
*CPP and pbtO*_*2*_*, %GMT above/below threshold*							
CPP < 60 mmHg (%)	4 (2–8)	4 (2–7)	5 (2–10)	***0.01***	4 (2–7)	6 (3–15)	** < *0.001***
CPP < 60 mmHg and pbtO_2_ < 15 mmHg (%)	0.1 (0.0–0.9)	0.1 (0.0–0.5)	0.2 (0.0–1.3)	***0.02***	0.1 (0.0–0.6)	0.3 (0.0–2.6)	***0.007***
CPP < 60 mmHg and pbtO_2_ > 15 mmHg (%)	2 (1–5)	2 (1–4)	2 (1–5)	0.70	2 (1–4)	3 (1–9)	0.07
CPP > 60 mmHg and pbtO_2_ < 15 mmHg (%)	1 (0–9)	1 (0–8)	2 (0–9)	0.45	1 (0–9)	2 (0–9)	0.88
*∆CPPopt and pbtO*_*2*_*, %GMT above/below threshold*							
∆CPPopt < −5 mmHg (%)	28 (22–37)	27 (20–32)	31 (24–42)	***0.003***	27 (20–32)	39 (26–52)	** < *0.001***
∆CPPopt < −5 mmHg and pbtO_2_ < 15 mmHg (%)	0.3 (0.0–3.3)	0.2 (0.0–1.9)	0.5 (0.0–4.6)	0.0503	0.2 (0.0–2.4)	0.6 (0.0–5.5)	0.06
∆CPPopt < −5 mmHg and pbtO_2_ > 15 mmHg (%)	19 (11–24)	19 (11–24)	18 (9–24)	0.55	18 (11–24)	21 (12–27)	0.16
∆CPPopt > −5 mmHg and pbtO_2_ < 15 mmHg (%)	1 (0–5)	1 (0–4)	0 (0–6)	0.92	1 (0–6)	1 (0–4)	0.31

Bold and italics indicate statistical significance

The variables in the table were described as median values (IQR). The Mann–Whitney *U* test was used to compare pbtO_2_ in the different outcome groups

*CPP* cerebral perfusion pressure, *CPPopt* optimal CPP, *ICP* intracranial pressure, *PbtO*_*2*_ partial brain tissue oxygenation, *PRx* pressure reactivity index, *∆CPPopt* CPP-CPPopt

### Multiple logistic regressions of unfavourable outcome and mortality

In a multiple logistic regression with unfavourable outcome as the dependent variable (Table 4), a higher age, lower GCS, and a higher %GMT of pbtO_2_ below 15 mmHg were independently associated with a higher rate of unfavourable outcome, whereas the %GMT of ICP above 20 mmHg, PRx above 0.30, and CPP below 60 mmHg were not associated with outcome. In a similar outcome regression, when CPP below 60 mmHg was replaced with the autoregulatory target ∆CPPopt < −5 mmHg, a higher age, lower GCS, and higher %GMT of ∆CPPopt < −5 mmHg were associated with a higher rate of unfavourable outcome, whereas %GMT of ICP above 20 mmHg, PRx above 0.30, and pbtO_2_ below 15 mmHg were not associated with outcome. In a multiple logistic regression with mortality as the dependent variable, a higher age, lower GCS, higher %GMT of ICP above 20 mmHg, and PRx above 0.30 were associated with mortality, whereas the %GMT of CPP below 60 mmHg and pbtO_2_ below 15 mmHg were not. In a similar outcome regression, when CPP below 60 mmHg was replaced with the autoregulatory target ∆CPPopt < −5 mmHg, a higher age, higher %GMT of ICP above 20 mmHg, PRx above 0.30, and ∆CPPopt < −5 mmHg were associated with a mortality, whereas lower GCS and pbtO_2_ below 15 mmHg were not.

**Table 4:** Cerebral physiological insults in relation to unfavourable outcome and mortality—multiple logistic outcome regressions

Variables	Unfavourable outcome		Mortality	
	OR (95% CI)	*p*	OR (95% CI)	*p*
*Absolute CPP insults*				
Age (years)	1.03 (1.01–1.05)	**< *0.001***	1.04 (1.01–1.06)	***0.004***
GCS (scale)	0.84 (0.77–0.93)	**< *0.001***	0.88 (0.78–0.99)	***0.04***
ICP > 20 mmHg (%)	1.01 (0.99–1.02)	0.29	1.03 (1.01–1.05)	***0.009***
PRx > 0.30 (%)	1.02 (1.00–1.04)	0.06	1.05 (1.02–1.07)	**< *0.001***
CPP < 60 mmHg (%)	1.04 (0.99–1.09)	0.09	1.04 (0.99–1.10)	0.10
PbtO_2_ < 15 mmHg (%)	1.02 (1.00–1.04)	***0.048***	1.00 (0.97–1.02)	0.77
*Autoregulatory CPPopt insults*				
Age (years)	1.03 (1.01–1.05)	**< *0.001***	1.03 (1.01–1.06)	***0.006***
GCS (scale)	0.85 (0.78–0.93)	**< *0.001***	0.91 (0.80–1.01)	0.08
ICP > 20 mmHg (%)	1.01 (0.99–1.02)	0.32	1.02 (1.00–1.04)	***0.02***
PRx > 0.30 (%)	1.02 (1.00–1.04)	0.09	1.04 (1.02–1.07)	**< *0.001***
∆CPPopt < −5 mmHg (%)	1.03 (1.00–1.06)	***0.03***	1.05 (1.02–1.09)	***0.002***
PbtO_2_ < 15 mmHg (%)	1.02 (1.00–1.04)	0.07	0.99 (0.97–1.02)	0.59

Bold and italics indicate statistical significance

CPP models, favourable outcome (AUROC (95% CI) = 0.76 (0.70–0.82), AIC = 270, Nagelkerke = 0.26) and mortality (AUROC (95% CI) = 0.83 (0.76-0.90), AIC = 181, Nagelkerke = 0.40)

CPPopt models, favourable outcome (AUROC (95% CI) = 0.76 (0.70–0.82), AIC = 266, Nagelkerke = 0.27) and mortality (AUROC (95% CI) = 0.84 (0.78-0.91), AIC = 173, Nagelkerke = 0.44)

*AIC* akaike information criterion, *CI* confidence interval, *CPP* cerebral perfusion pressure, *CPPopt*  optimal CPP, *GCS* Glasgow Coma Scale, *ICP* intracranial pressure, *OR* odds ratio, *PbtO*_*2*_ partial brain tissue oxygenation, *PRx* pressure reactivity index

## Discussion

In this study, including 239 TBI patients, we found that pbtO_2_ below 25 mmHg was fairly common and there was a gradual transition towards unfavourable outcome for longer periods of time below this value. The association of %GMT or dose with pbtO_2_ below threshold and outcome was most pronounced below 15 mmHg, although such insults overall were rare (GMT < 5%). Furthermore, low pbtO_2_ without concurrent disturbances in the global cerebral physiological variables did not correlate with worse outcome. The association between low pbtO_2_ and outcome was more pronounced when any of the other global variables, i.e. ICP, PRx, CPP, or ∆CPPopt, was concurrently deranged. This supports the notion that low pbtO_2_ in combination with disturbances in global cerebral physiological variables indicate detrimental spatially widespread ischaemic hypoxia. However, pbtO_2_ is a focal measure and normal values do not exclude ischaemic hypoxia elsewhere.

First, low pbtO_2_ was associated with unfavourable outcome and to some extent with mortality (chi-square analyses), consistent with several previous studies [8–10]. This corroborates that although the pbtO_2_-probe is focally restricted to monitor a small brain region, it conveys clinically important information that is relevant for long-term recovery. It appeared that the %GMT and dose per hour below certain pbtO_2_ thresholds (e.g. 15 mmHg) were more strongly associated with outcome than median/mean values. This was expected considering the crude nature of median/mean values to capture brief, but dangerously low values.

Second, regarding mmHg thresholds, in the statistical analyses, including the percentage of GMT, dose per hour, and the insult intensity/duration heatmap, there was a transition towards unfavourable outcome when pbtO_2_ went below 25 mmHg. The transition started slightly higher for the dose concept, which was expected since it also takes into account the cumulative magnitude (mmHg below the threshold being explored). The transition around 25 mmHg may be considered a relatively high value, since pbtO_2_ has similarly been shown to be around 20–25 mmHg in otherwise healthy patients undergoing elective neurosurgery [34]. However, it is possible that the acutely injured brain following TBI indeed requires a normal to somewhat higher pbtO_2_. Previous, smaller (*n* ≈ 100), TBI studies have reported similar thresholds for outcome associations as compared to this study. The threshold for mortality/survival has been reported to fall between 10 and 29 mmHg [7, 10] and for favourable/unfavourable outcome more narrowly between 15 and 20 mmHg [6, 7, 9]. In this study, we did not only look at the absolute values of pbtO_2_, but also explored the potential time-dependent effect of exceeding an insult intensity. This was done both by investigating the accumulated burden of exceeding a certain threshold (%GMT and dose of pbtO_2_ below e.g. 15 mmHg) and for specific durations for individual insult intensities (e.g. pbtO_2_ below 15 mmHg for exactly 15 min in the heatmap). The heatmap was particularly important to visualise the role of pbtO_2_ deteriorations, since it is acknowledged that pbtO_2_ trends may exhibit different patterns [8], such as stable high or stable low values, initially high with gradual deterioration, or mainly high values with frequent brief drops. As low pbtO_2_ is only occasionally associated with high ICP and low CPP [6, 9], it is challenging to determine if some of these deteriorations reflect detrimental pathophysiology or are merely artefacts. Our heatmap then suggested that pbtO_2_ deteriorations were clinically relevant as they were associated with poor outcome. Furthermore, the heatmap indicated that what defines a dangerously low pbtO_2_ could depend on the duration of the insult. Altogether, in comparison to the traditional fixed 20 mmHg pbtO_2_ threshold [13], our results indicate that it is desirable not to let pbtO_2_ go below 15 mmHg even for shorter durations and preferably to keep it above 25 mmHg.

Third, it was clear that events of pbtO_2_ below 15 mmHg in combination with disturbances in any of the global physiology-related variables ICP, PRx, CPP, or ∆CPPopt, were rare and only occurred in less than 1% in most patients. The limited overlap between insults in global cerebral physiological variables and pbtO_2_ was to some extent expected, as there is a significant intra-individual spatial variability in CBF [35] and it has been argued that in severe TBI a focal cerebral ischaemia only overlaps with hypoxia to some extent [11, 12], which was also supported by the findings in part I of this study. In other words, the global perfusion-related variables were expected to be poor predictors of focal deficits in CBF and a focal CBF impairment was expected to be a poor predictor of focal hypoxia. Since pbtO_2_ was typically monitored in a non-eloquent brain area (right frontal lobe), an isolated pbtO_2_-insult would then also not be expected to have a major effect on a global outcome measure such as GOS. However, when both global cerebral physiological variables and focal pbtO_2_ were disturbed at the same time, it was more likely that the entire brain was affected by ischaemic hypoxia, which could explain why even though this was rare it correlated with worse outcome. Although such combined insults only occurred in median in less than 1% of monitoring time, more than 50% of the patients exhibited such combined deranged values at least at some point in the NCC, indicating that they were infrequent but detrimental. In this scenario, it is expected that therapies oriented towards CBF augmentation would be beneficial. Furthermore, low pbtO_2_ alone without concurrent disturbances in global cerebral physiological variables was not associated with worse outcome. Thus, it is possible that therapies targeted to improve isolated pbtO_2_ insults may have somewhat limited clinical value due to the reasons mentioned above. However, further insights in this matter will be revealed when the three ongoing RCTs on pbtO_2_-oriented management have been completed.

Fourth, despite that isolated pbtO_2_ insults were weakly associated with outcome, we found that %GMT of pbtO_2_ below 15 mmHg was independently associated with unfavourable outcome, even after adjustment for %GMT of ICP > 20 mmHg, PRx > 0.30, and CPP < 60 mmHg. We speculate that this finding reflects the times when low pbtO_2_ indicated cerebral decompensation of widespread ischaemic hypoxia due to hypoperfusion. However, this association was attenuated when CPP < 60 mmHg was replaced by the autoregulatory target ∆CPPopt < −5 mmHg, which might be an even stronger indicator of global cerebral hypoperfusion.

Fifth, there are now three ongoing phase III trials on the role of pbtO_2_-directed treatments in addition to ICP and CPP [13], which may reveal if such management translates into better neurological recovery. The protocols in these trials are based on a pbtO_2_ threshold at 20 mmHg; however, it is possible that considering both thresholds of pbtO_2_ intensity and duration could be an alternative approach, since values below 25 mmHg may be detrimental for the brain if the insults persist over longer time periods. In another, more individualised, approach, pbtO_2_-directed therapies could be guided by the concurrent use of microdialysis monitoring, since the latter could indicate when energy metabolic decompensation starts to occur due to pbtO_2_ deterioration for the individual patient [4]. Yet another approach would be to guide global pbtO_2_ therapies based on if such deteriorations translate into increases in blood-based biomarkers of global brain damage [36].

Lastly, the clinical implications of this study are that low pbtO_2_ carries clinically relevant information for outcome and pbtO_2_ appears to be a valuable multimodality monitoring tool for making patient prognosis and possibly guiding treatment. In the clinical interpretation of pbtO_2_, not only the absolute intensity level, but also the duration of exceeding certain threshold ranges should be taken into account, since lower intensity levels may be tolerated for a slightly longer time, whereas higher intensities may be very dangerous even for brief periods. The absolute pbtO_2_ level should also be viewed in relation to the global cerebral physiological variables ICP, PRx, CPP, and ΔCPPopt. If the focal pbtO_2_ measure and any of these global variables are deranged at the same time, this is a particularly ominous brain state and management should be devoted to control ICP and augment CPP/improve ΔCPPopt. The clinical implications of isolated pbtO_2_ disturbances are less clear and it remains to be determined if it adds any value to treat this condition by e.g. augmentation of CBF or arterial oxygen content.

### Strengths and limitations

The main strength of this manuscript was a relatively large cohort size (*n* = 239) of TBI patients with high-frequency data of ICP and pbtO_2_. We also used an extensive approach to study insult-thresholds from different angles. Particularly, the heatmaps visualised in a novel way how the effects of exceeding insult intensities may be time dependent and the importance of combined perfusion-related vs. isolated pbtO_2_ insults.

There were also some limitations. First, demographic and clinical data could not be retrieved in some cases. Second, previous studies indicate that pbtO_2_ measured close to focal traumatic lesions is lower than in normal-appearing tissue [8, 37]. In our study, the pbtO_2_ probe was typically inserted in the non-dominant frontal lobe while direct placement in lesions was avoided according to protocol. However, unfortunately, we were unable to access the radiological images to confirm this. This also prohibited us from proceeding with sub-analyses on the influence of pbtO_2_ from a peri-lesional vs normal-appearing probe location in relation to outcome. Third, this was a single-centre study with a selection of those patients who received both ICP- and pbtO_2_ monitoring. This limits the external validity of our findings. Fourth, the reliability of PRx and CPPopt has been questioned for EVD based ICP measurements and for patients with decompressive craniectomy. We attempted to address this issue with careful signal processing as explained in part I. Fifth, clinical outcome is a complex measure that is related to many factors, such as age, injury patterns, NCC treatments, and rehabilitation, which may have influenced the relative importance of pbtO_2_ derangements in relation to GOS. Sixth, the heatmaps were to some extent sensitive to noise, particularly for insults that were rare, which reduced the reliability of these areas in the plots. Seventh, we explored several pbtO_2_ thresholds; however, it was inevitable that they overlapped to some extent, since exceeding a certain value for a certain duration also implied that an even higher intensity might have been exceeded for some time during that period. Consequently, this makes it to some extent challenging to determine at what specific intensity and duration secondary brain injury occurred. Lastly, the data analysed in this retrospective study reflects more than just physiological or pathophysiological dynamics; it represents the intricate interplay of a dedicated NCC team striving to optimise brain physiology and physiological variables. The strength of their predictive prognostic values observed in the study are influenced not only by the patients' physiological responses but also by the collective efforts and expertise of the healthcare professionals involved in their care. Therefore, while the study findings may suggest a limited association between pbtO_2_ and outcomes, it is crucial not to interpret this as a justification for a laissez-faire approach. The proactive and tailored interventions implemented by NCC teams remain essential in optimising brain function and ensuring the best possible outcomes for patients.

## Conclusions

In TBI patients treated in NCC, pbtO_2_ below 20–25 mmHg was fairly common and there was a gradual transition towards unfavourable outcome for longer periods of time below these values and even more pronounced below 15 mmHg. Interestingly, pbtO_2_ below 15 mmHg was particularly associated with poor outcome when ICP, PRx, CPP, or ∆CPPopt were also deranged, but not when these variables were controlled. This suggests that pbtO_2_ should be considered in combination with the global physiology-related variables to indicate detrimental ischaemic hypoxia. However, isolated pbtO_2_ insults may have somewhat limited clinical value.

## Data Availability

Data are available upon reasonable request.

## Abbreviations

ABP: Arterial blood pressure
AIC: Akaike information criterion
CBF: Cerebral blood flow
CPP: Cerebral perfusion pressure
CPPopt: Optimal CPP
GMT: Good monitoring time
GOS: Glasgow Outcome Scale
ICP: Intracranial pressure
IQR: Interquartile range
NCC: Neurocritical care
PbtO_2_: Partial brain tissue oxygenation
PRx: Pressure reactivity index
SE: Standard error
TBI: Traumatic brain injury
